# Supplementary material for: Morphological and Molecular Identification of Plant Pathogenic Fungi Associated with Dirty Panicle Disease in Coconuts (Cocos nucifera) in Thailand
Source: J Fungi (Basel). 2022 Mar 23;8(4):335. doi: 10.3390/jof8040335 (PMC9029170; doi:10.3390/jof8040335)
Supplement: Supplementary file 1 [file jof-08-00335-s001.zip › Supplementary Figure S1.pdf]

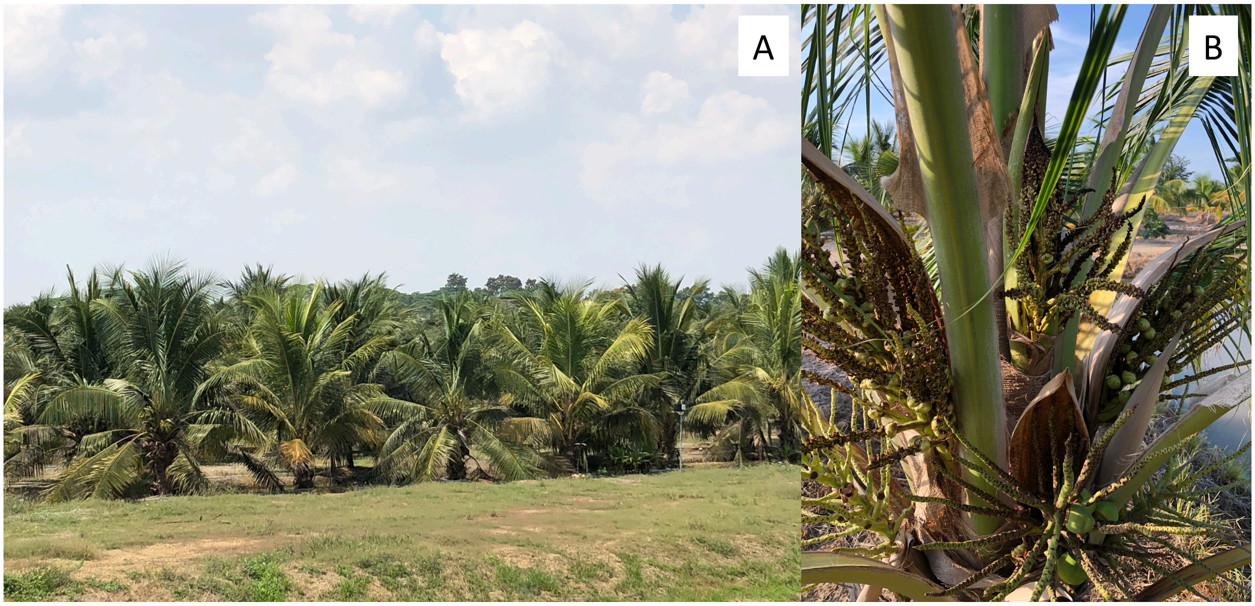

Supplementary Figure S1. A) KU-BEDO Coconut BioBank on the campus of Kasetsart University, Kamphagen Saen, Nakhon Pathom, Thailand. The plantation is 4.8 hectares in size and includes 770 coconut trees. B) Dirty panicle disease observed in coconut plants grown in the biobank.
