# Supplementary material for: Morphological and Molecular Identification of Plant Pathogenic Fungi Associated with Dirty Panicle Disease in Coconuts (Cocos nucifera) in Thailand
Source: J Fungi (Basel). 2022 Mar 23;8(4):335. doi: 10.3390/jof8040335 (PMC9029170; doi:10.3390/jof8040335)
Supplement: Supplementary file 1 [file jof-08-00335-s001.zip › Supplementary-Table S1.pdf]

Supplementary Table S1. Gene specific primers used for DNA amplification and sequencing.

Primers	Sequences (5″- 3′)	Product size (bp)	Reference
ITS1	TCCGTAGGTGAACCTGCGG	500	White et al. (1990)
ITS4	TCCTCCGCTTATTGATATGC		
fRPB2-5f	GAYGAYMGWGATCAYTTYGG	800	Liu et al. (1999)
fRPB2-7cr	CCCATRGCTTGYTTRCCCAT		
Tef1-728F	CATCGAGAAGTTCGAGAAGG	250	Carbone & Kohn (1999)
Tef1-986R	TACTTGAAGGAACCCTTACC		
GPD1	CAACGGCTTCGGTCGCATTG	600	Larsson et al. (1993)
GPD2	GCCAAGCAGTTGGTTGTGC		
